# EPO enhances the protective effects of MSCs in experimental hyperoxia-induced neonatal mice by promoting angiogenesis

**DOI:** 10.18632/aging.101937

**Published:** 2019-04-29

**Authors:** Chao Sun, Shanshan Zhang, Jue Wang, Wen Jiang, Qian Xin, Xiaojing Chen, Zhaohua Zhang, Yun Luan

**Affiliations:** 1 Central Research Laboratory, The Second Hospital of Shandong University, Jinan 250033 PR China; 2 Department of Emergency, The Second Hospital of Shandong University, Jinan 250033 PR China; 3 Department of Pediatrics, The Second Hospital of Shandong University, Jinan 250033 PR China; * Equal contribution

**Keywords:** BPD, MSCs, EPO, angiogenesis, SDF-1/CXCR4

## Abstract

Bronchopulmonary dysplasia (BPD) is the most common type of chronic lung disease in infancy; however, there is no effective treatment for it. In the present study, a neonatal mouse BPD model was established by continuous exposure to high oxygen (HO) levels. Mice were divided randomly into 5 groups: control, BPD, EPO, MSCs, and MSCs+EPO. At 2 weeks post-treatment, vessel density and the expression levels of endothelial growth factor (VEGF), stromal cell-derived factor-1 (SDF-1), and its receptor C-X-C chemokine receptor type 4 (CXCR4) were significantly increased in the MSC+EPO group compared with the EPO or MSCs group alone; moreover, EPO significantly enhanced MSCs proliferation, migration, and anti-apoptosis ability *in vitro.* Furthermore, the MSCs could differentiate into cells that were positive for the type II alveolar epithelial cell (AECII)-specific marker surfactant protein-C, but not positive for the AECI-specific marker aquaporin 5. Our present results suggested that MSCs in combination with EPO could significantly attenuate lung injury in a neonatal mouse model of BPD. The mechanism may be by the indirect promotion of angiogenesis, which may involve the SDF-1/CXCR4 axis.

## INTRODUCTION

Bronchopulmonary dysplasia (BPD) is a chronic respiratory disease in preterm infants requiring mechanical ventilation and oxygen therapy [1], and is characterized by restricted lung growth and subdued alveolar and blood vessel development; the structural development of the alveoli is blunted as a consequence of inflammation and oxygen toxicity [2, 3]. To date, there is no effective therapy for preventing or treating lung injury, and new therapies are urgently needed. A large number of studies [4–6] have shown that stem cell-based therapies, such as mesenchymal stem/stromal cells (MSCs), are promising approaches in preclinical models for the prevention and/or treatment of BPD and other major sequelae of preterm birth. However, there are still some crucial problems that largely restrict the efficacy of these treatments, such as cell survival, homing, and differentiation [7].

Erythropoietin (EPO) is a glycoprotein hormone produced primarily by the adult kidney [8, 9]. Over the last decade, EPO has been shown to be an important cytoprotective cytokine against various forms of stress in many organs, including pulmonary disease [10–12], and the mechanism is through the repair of alveolar structure, enhancement of angiogenesis, and suppression of fibrosis [13, 14]. The proper formation of the microvascular system is necessary for normal alveolar development, and an abnormal microvascular system has been observed in the lung of infants with BPD and in BPD-like animal models. Vascular endothelial growth factor (VEGF) plays an essential role in stimulating angiogenesis and the survival of endothelial cells [15]. Reports have showed that VEGF expression and pulmonary capillary density are significantly decreased in the lungs of BPD patients and animal models [16, 17]. In addition, increased VEGF expression inhibits high oxygen (HO)-induced alveolar disruption [18]. However, the influence of combination therapy with EPO and MSCs on angiogenesis in BPD and the underlying mechanism are not fully clear; therefore, the present study was performed to examine these issues.

## RESULTS

### Characterization of MSCs

Characteristic immunoreactivity for cell surface markers was detected using FACS analysis with rat monoclonal anti-mouse antibodies. The cells were positive for the expression of the surface markers CD44, CD90, and CD106, but negative for the expression of the hematopoietic markers CD34, CD45, and CD117.

### Effect of MSCs+EPO combination therapy on body weight and lung injury

**Figure 1:**
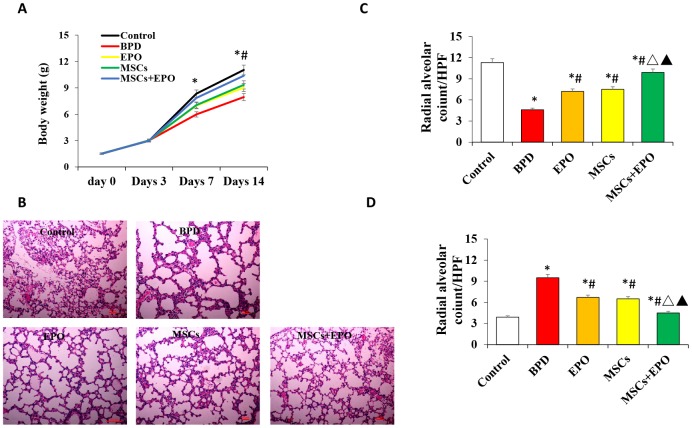
**Effect of MSCs+EPO on body weight and lung injury.** Experimental animals were continuously exposed to high oxygen environment for14 days to established BPD mode. MSCs and EPO were injected at 1h before and 7d after high oxygen exposure. (**A**) The average body weight was measured at 3-,7- and 14- day post-operation in each group. (**B**) Lung histopathology was detected by H&E staining(×100 magnification). (**C**) A morphometric analysis was conducted using performing radial alveolar counts (RAC) and, (**D**) A morphometric analysis of the alveolar septum thickness. Data are presented as the mean ± SD. ^*^P < 0.05 compared with the control group; ^#^*P* < 0.05 compared with the BPD group; ^△^*P*<0.05 compared with the EPO; ^▲^*P*<0.05 compared with the MSCs group.

The body weight of neonatal mice was measured at 3, 7, and 14 days post-treatment. As shown in Figure 1A, average body weight was significantly increased in the EPO, MSCs and MSCs+EPO groups as compared with the BPD model group; moreover, body weight was increased more in the MSCs+EPO group than in the MSCs or EPO group alone(*P* < 0.05). Hematoxylin and eosin-stained sections of lung tissue demonstrated that alveolar structure was markedly abnormal after neonatal mice were exposed to high oxygen for 14 days (Figure 1B), furthermore, degree of alveolarization measured by radial alveolar counts (Figure 1C) and alveolar septum thickness (Figure 1D) were significantly improved in the MSCs, EPO and MSCs+EPO groups compared with BPD group, especially in the MSCs+EPO group (*P* < 0.05).

### Effect of MSCs+EPO combination therapy on vessel density and cell apoptosis

**Figure 2:**
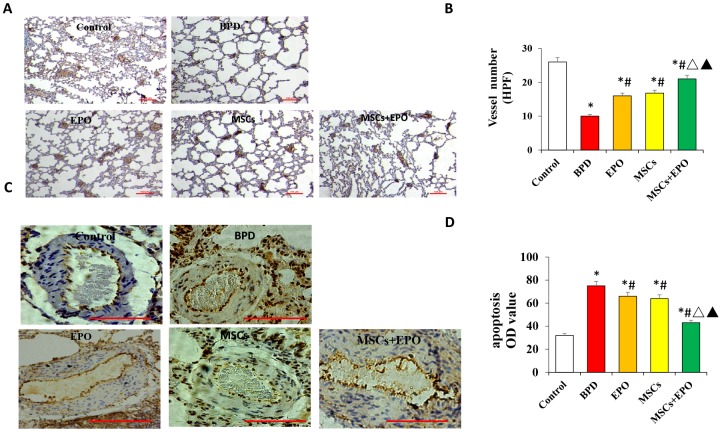
**Vascular density and apoptosis detection by immunohistochemistry and qRT-PCR *in vivo*.** (**A**) PECAM-1 staining of microvascular endothelial cells (×100 magnification). (**B**) Comparative analysis of vascular density in lung tissue. (**C**) TUNEL analysis of pulmonary vascular cells apoptosis in lung tissue. (**D**) Comparative analysis of the OD value of TUNEL-positive cells in each group. Data are presented as the mean ± SD. ^*^P < 0.05 compared with the control group; ^#^*P* < 0.05 compared with the BPD group; ^△^*P*<0.05 compared with the EPO; ^▲^*P*<0.05 compared with the MSCs group.

The number of new vessels was significantly increased in both treatment groups compared with the BPD group (*P* < 0.05, Figure 2A and 2B).

Moreover, the number of new vessels was higher in the MSCs+EPO group than in the MSCs or EPO group alone (*P* < 0.05). Apoptosis of pulmonary vascular cells was also determined inlung tissue. As shown in Figure 2C and 2D, TUNEL-positive cells were significantly decreased in the EPO, MSCs and MSCs+EPO groups as compared with the BPD group, especially in the MSCs+EPO group (*P* < 0.05).

### Effect of combination therapy on cell proliferation and identification *in vivo*

**Figure 3:**
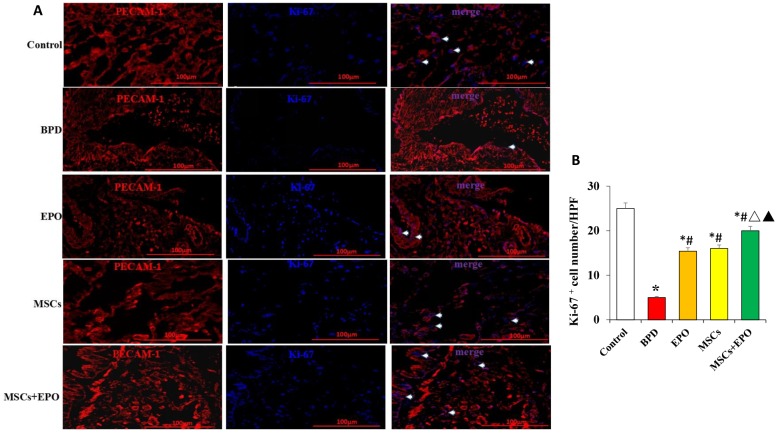
**Immunofluorescence analysis of the proliferative activity of microvascular endothelium *in vivo*.** (**A**) Ki-67 (blue) and PECAM-1 (red) double staining using a light microscope at ×400 magnification. (**B**) Comparative analysis of the OD value in each group. Data are presented as the mean ± SD. ^*^P < 0.05 compared with the control group; ^#^*P* < 0.05 compared with the BPD group; ^△^*P*<0.05 compared with the EPO; ^▲^*P*<0.05 compared with the MSCs group.

**Figure 4:**
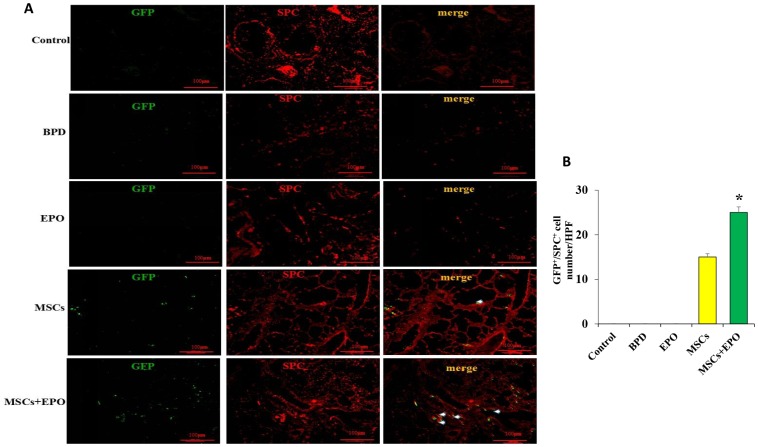
**Identification of transplanted MSCs in lung tissue *in vivo*.** (**A**) Transplanted cells were marked with GFP and the AECII-specific marker SP-C in red at ×200 magnification. (**B**) Comparative analysis of cell number in the MSCs and MSCs+EPO groups. Data are presented as the mean ± SD. ^*^P < 0.05 compared with the MSCs group.

Double immunofluorescence staining for Ki-67 and PECAM-1 was used to determine the proliferative activity of microvascular endothelium. The optical density (OD) value of representative sections was significantly increased in the MSCs+EPO group compared with the MSCs or EPO group alone (Figure 3A and 3B). The transplanted cells were tracked through GFP; MSCs were marked with green and the AECII-specific marker SP-C was stained red, and the results indicated a higher differentiation rate in the MSCs+EPO group than in the MSCs group (*P* < 0.05, Figure 4A and 4B).

### Effect of MSCs+EPO combination therapy on the expression of CXCR4, SDF-1, and VEGF *in vivo*

**Figure 5:**
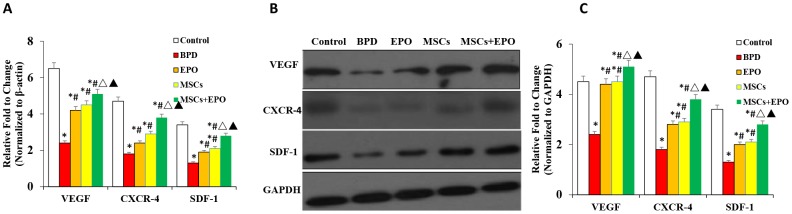
**Analysis of mRNA and protein expression of CXCR4, SDF-1, and VEGF in lung tissue.** (**A**) mRNA levels of CXCR4, SDF-1, and VEGF were evaluated by qRT-PCR. (**B**) Protein expression analysis of CXCR4, SDF-1, and VEGF by western blotting. Data are presented as the mean ± SD. ^*^P < 0.05 compared with the control group; ^#^*P* < 0.05 compared with the BPD group; ^△^*P*<0.05 compared with the EPO; ^▲^*P*<0.05 compared with the MSCs group.

qRT-PCR and western blot analyses indicated that the mRNA and protein levels of CXCR4, SDF-1, and VEGF were significantly decreased in the lung tissue of three treatment groups when compared with the BPD group; moreover, a greater improvement was observed in the MSCs+EPO group compared with the MSCs or EPO group alone (*P* < 0.05, Figure 5A–5C).

### Effect of EPO on MSC proliferation, migration, and apoptosis *in vitro*

**Figure 6:**
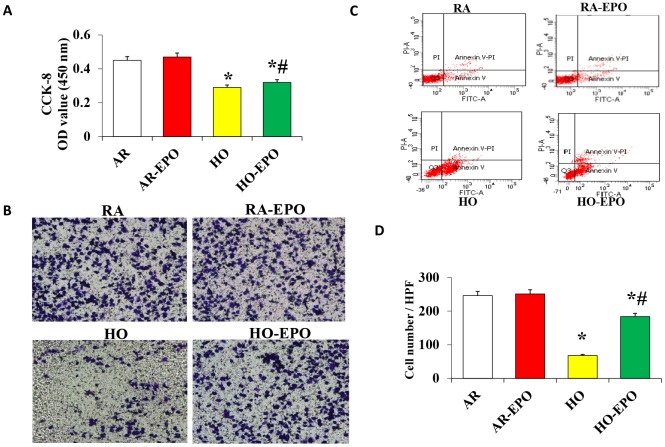
**Analysis of MSC proliferation, migration, and apoptosis *in vitro*.** (**A**) Cell proliferation was detected by the colorimetric CCK-8 assay. (**B**) Cell migration was detection by a Transwell assay *in vitro*. (**C**) Cell apoptosis was analyzed by flow cytometry using annexin V binding and PI staining. Data are presented as the mean ± SD. ^*^P < 0.05 compared with the control group; #P < 0.05 compared with the HO-MSCs group.

The proliferation and migration of MSCs were significantly decreased in the high oxygen (HO) group compared with the room air (RA, control) group (Figure 6A and 6B); however, proliferation and migration were significantly increased in the cells in the HO environment with complete medium containing 2 U/mL EPO. Analysis of apoptosis by flow cytometry showed that there was no difference between the RA and RA-EPO groups (6.64% vs. 6.87%, respectively), but apoptosis was increased (45.16%) when the cells were exposed to an HO environment than to an RA environment (*P* < 0.05); moreover, a clearly lower rate of apoptosis was observed when the complete medium contained 2 U/mL EPO (26.58%, *P* < 0.05, Figure 6C).

## DISCUSSION

This study indicated that combination treatment with MSCs and EPO could significantly repair HO-induced alveoli dysplasia damage. Moreover, the density of new vessels and the expression of VEGF, SDF-1, and its receptor CXCR4 were significantly higher in the MSCs+EPO group than in the MSCs or EPO group alone. Furthermore, our data confirmed that EPO improved the proliferation, migration, and anti-apoptosis ability of MSCs *in vitro* and *in vivo*.

Neonatal chronic lung disease, known as BPD, remains a serious complication of prematurity despite advances in the treatment of extremely low birth weight infants, and there is no effective treatment for this disease. Studies have shown that MSC transplantation could repair alveolar damage and promote angiogenesis in experimental HO neonatal rat models [19], suggesting it may become a novel treatment for BPD [20, 21]. Although transplanted MSCs can migrate to lung tissue in BPD animals and patients [22–24], their underlying role and mechanism have not been identified [25], and some crucial problems remain that largely restrict their protective role, such as cell survival, homing, and differentiation.

EPO is a glycoprotein hormone produced primarily by the adult kidney that can inhibit apoptosis, inflammation, and oxidation. Over the last decade, EPO has emerged as an important cytoprotective cytokine against various forms of stress. Recently, EPO was found to have new pharmacological activity; for example, it can reduce the incidence rate of BPD in premature infants, especially in the first 4 weeks [26], and it can stimulate the differentiation and proliferation of erythroid progenitor cells [27]. In our previous study, we showed that EPO promotes the therapeutic effect of MSCs in BPD animals [13], but the mechanism was not clear. Our present study indicated that EPO enhanced MSC proliferation and migration, induced a remarkable decrease in the rate of apoptosis in an HO environment *in vitro*. This may be the mechanism by which EPO promotes the repair of BPD lung injury by MSCs.

An abnormal microvascular system is the main cause of BPD [16, 28, 29]. As is well known, the appropriate form of pulmonary microvascular is necessary for the normal development of alveolar structure [17, 30]. Pulmonary capillary density and the expression of VEGF and its receptor are significantly decreased in BPD [16, 17]. Therefore, we think that the promotion of blood vessel development is one of the key steps in the prevention and treatment of BPD with MSCs, and the findings of the present study support this hypothesis. Our results showed that the number of new vessels and VEGF expression were significantly increased in the MSCs+EPO group compared with the MSCs group *in vivo*. Our previous study [14] showed that EPO did not enhance the ability of MSCs to differentiate into vascular endothelial cells *in vivo*. Therefore, these results suggest that the underlying mechanism for the improvement of angiogenesis by combination treatment is indirectly through a paracrine effect.

AECII synthesize and secrete a surface-active material to adjust and maintain alveolar surface tension to repair injured type I epithelium in mammalian lungs [31–32]. However, the role of AECII is largely unknown. The differentiation of MSCs in the lung is still controversial, and some researchers have reported that MSCs can differentiate into AECI [33], while other reports showed that MSCs play a protective role in pulmonary fibrosis induced by bleomycin and mainly differentiate into AECII [34, 35]. In this study, our results suggested that MSCs could differentiate into cells positive for the AECII-specific marker SP-C, but negative for the AECI-specific marker AQP5.

Poor survival and directional homing limit the therapeutic effect of transplanted MSCs. SDF-1 is a member of the CXC chemokine family that plays a key role in tissue repair and angiogenesis. In combination with its receptor CXCR4, SDF-1 can enhance the proliferation, migration, and anti-apoptotic ability of MSCs, and can also increase the survival rate of transplanted cells [36, 37] and promote the homing and differentiation of MSCs [38, 39]. Angiogenesis is a complex physiological process that involves individual regulation and cross-talk of angiogenic factors [40]. The SDF-1/CXCR4 axis is involved in pulmonary vascular disease by mediating the accumulation of c-kit+ cells [41]. In order to identify the mechanism by which EPO promotes MSC survival, homing, and angiogenesis, we detected the expression levels of VEGF, SDF-1, and CXCR4. The mRNA and proteins levels of VEGF, SDF-1, and CXCR4 were significantly increased in the MSCs+EPO combination group compared to the MSCs or EPO group alone *in vivo*. Recently studies showed that MSCs transplantation in the treatment of BPD mainly through paracrine role, and exosomes released from MSC was the responsible therapeutic vector for the “main” therapeutic effects afforded by MSCs [13]. Considering the saccular stage of lung development and the lung development process, choose the best point as a treatment time seems to be very necessary. Those can not be clarified in the present study and would need further investigations. Taken together, our findings give us at least two insights about the mechanism by which EPO enhances the repair of HO injury by MSCs. The first was the improvement of MSC proliferation, migration, and homing, and the second was the promotion of angiogenesis through the SDF-1/CXCR4 axis.

Collectively, our experiments suggested that MSC and EPO combination therapy could significantly attenuate alveoli dysplasia injury in an HO-induced neonatal mouse model of BPD. The mechanism may be via the improvement of MSC proliferation, migration, and anti-apoptosis ability and the indirect promotion of angiogenesis, which may involve the SDF-1/CXCR4 axis.

## MATERIALS AND METHODS

### MSC culture

C57BL/6-green fluorescent protein (GFP) transgenic mice (6–8 weeks old) were bought from the experimental animal center of the Fourth Military Medical University (Xian, China). MSCs were isolated from tibias and femurs using a whole bone marrow culture method as described in our previously study [14]. Characteristic immunoreactivity for cell markers was detected using fluorescence-activated cell sorting (FACS) analysis (FACScan LSRFortessa; BD Bioscience, Franklin Lakes, NJ).

### BPD model and cell transplantation

Neonatal C57BL/6 mice (24 h old; 1–2 g) were obtained from the Animal Experiment Center of Shandong University (Shandong, China). All animal procedures were approved by the animal ethics committee. BPD model was established as previously described with some modifications [13, 14], briefly, BPD model was established through placed the pups animals in Plexiglas chamber, in which the oxygen concentration was maintained at a FiO2=0.21 (normoxia) or FiO2=0.60 (hyperoxia) for 21 days. Exposure to hyperoxia was continuous, and mice were maintained in a hypoxic environment, with a brief interruption for animal care (less than 10 min/day). Cultivated MSCs (1–5 × 10^6^) in 50 μl phosphate-buffered saline (PBS) was injected via intravenous administration or/and 5000U/kg recombinant human EPO (Sigma-Aldrich, St. Louis, MO) by intraperitoneal injection respectively at 1 h before and at 7 days after HO exposure.

### Histology and immunohistochemical analysis

At 2 weeks post-treatment, the lungs were harvested and fixed in 4% paraformaldehyde for 24 h. The tissue was embedded in paraffin and cross-sections were cut with a microtome at 4–5 μm (Leica RM226; Leica Microsystems, Heidelberg Germany). Hematoxylin and eosin (Baso Biotechnology, Shenzhen, China) staining was used to analyze radial alveolar counts and alveolar septum thickness. A total of five counts were performed for each animal; the average count was determined from five randomly selected high-power fields (magnification ×100).

Immunohistochemistry was used to evaluate microvascular growth. Briefly, the tissue was incubated with a primary anti-platelet endothelial cell adhesion molecule 1 (PECAM-1) antibody (ab28364; Abcam, Cambridge, UK) overnight or a nonspecific IgG antibody for 1 h at room temperature, and then a 2-step Plus® poly-horseradish peroxidase anti-mouse/rabbit IgG detection system (PV-9000; ZSGB-Bio Co., Beijing, China) was used.

To measure the apoptosis degree of the pulmonary vascular cells, terminal dUTP nick end-labeling (TUNEL) assay was performed. Briefly, fixed tissue sections were incubated at 37°C for 1 h with 50μl TUNEL-reaction mixture (including 5 μl terminal desoxynucleotidyl-transferase (TdT) and 45 μl dUTP-biotin) after proteinase K (20 μg/ml in PBS) treatment. Subsequently, the 3,3'- diaminobenzidine (DAB) dye was added to visualize the antibodies, and following washing of the tissue sections with phosphate-buffered saline (PBS) solution. The average of the 10 high-power fields (hpf) was randomly selected, and the apoptosis cell was defined as the number of vessels/hpf in the heart.

The proliferative activity of microvascular endothelium was determined by double immunofluorescence staining with rabbit anti-mouse PECAM-1 and Ki-67 antibodies, and differentiation was detected by staining with a rabbit anti-mouse type II alveolar epithelial cell (AECII)-specific marker surfactant protein-C (SP-C) antibody and an AECI-specific marker aquaporin 5 (AQP5) antibody *in vivo*. The cryosections were first blocked with 5% goat serum for 30 min and incubated with primary antibodies overnight at 4°C, which was followed by 1-h incubation in the dark with tetramethylrhodamine isothiocyanate (ZF-0316; ZSGB-Bio Co., Beijing, China) and a DyLight 350-conjugated (A23020; Abbkine, Wuhan, China) goat anti-rabbit IgG (H+L) secondary antibody at a dilution of 1:200.

Immunofluorescent images were taken with an Eclipse 90i microscope (Nikon, Tokyo, Japan). Staining was quantified using Image Pro Plus 6.0 image analysis software (Media Cybernetics, Rockville, MD) as described previously [13]. All experiments were performed by two examiners blinded to treatment assignment.

### Quantitative real-time reverse transcription PCR analysis

The mRNA levels of VEGF, C-X-C chemokine receptor type 4 (CXCR4), and stromal cell-derived factor-1 (SDF-1) were detected by quantitative real-time reverse transcription PCR (qRT-PCR). Total RNA was extracted from lung tissue using TRIzol® reagent (Invitrogen, Carlsbad, CA) according to the manufacturer’s instructions. The following primers were used: CXCR4 forward, 5′-GGCTGACCTCCTCTTTGT-3′ and reverse, 5′-GTTTCCTTGGCCTTTGAC-3′; SDF-1 forward, 5′-CCCTGCCGATTCTTTGAC-3′ and reverse, 5′-GTCCTTTGGGC TGTTGTG-3′; VEGF forward, 5′-CTGCTCTCCTGGGTGCATTG-3′ and reverse, 5′-ACTCCTGGAAGATGTCCACCA-3′; and GAPDH forward, 5′-ACTCTGGCAA AGTGGATATTGTCG-3′ and reverse, 5′-CAGCATCACCCCATTTGATG-3′. The cycling conditions comprised of 10 min polymerase activation at 95°C and 40 cycles at 95°C for 15 s and 60°C for 60 s. The values of the genes were first normalized against GAPDH, and then compared with controls.

### Western blot analysis

Protein expression was detected by western blot analysis. Total protein was electrophoresed on 4–20% gradient sodium dodecyl sulfate-polyacrylamide gel electrophoresis gels and transferred to a nitrocellulose membrane. Anti-CXCR4, anti-SDF-1, and anti-VEGF primary antibodies were used, and the membrane was incubated at 37°C for 1 h with a secondary goat anti-rabbit IgG antibody (Boshide, Inc., Shanghai, China). Immunoreactions were visualized using an electrochemiluminescence kit (Amersham Biosciences, Little Chalfont, UK) according to the manufacturer’s instructions.

### MSC proliferation analysis *in vitro*

The proliferation of MSCs was analyzed using a Cell Counting Kit-8 (CCK-8) assay. Briefly, 1.0 × 10^4^ subconfluent MSCs were detached by trypsinization, resuspended, and seeded into 96-well plates in a room air (RA, 21% O_2_ and 5% CO_2_) or HO (95% O_2_ and 5% CO_2_) environment. After 24-h incubation, the CCK-8 reagent was added to each well for 3 h at 37°C. Absorbance was measured at 450 nm using a microplate reader.

### MSC migration analysis *in vitro*

Migration assays were performed using Transwell cell culture chambers. Briefly, 8-μm pore polycarbonate filters were placed between the upper and lower chamber pore filters (Corning Life Sciences). A total of 5.0 × 10^4^ cells in 200 μL medium containing 1% (v/v) fetal bovine serum (FBS) were plated in the upper chamber, and the lower chamber was filled with complete medium containing 10% (v/v) FBS with or without 2 U/mL EPO and cultured at 37°C for 24 h. The upper surface of the Transwell membrane was wiped clean with a cotton swab, and the cells that had migrated to the lower surface were fixed and stained with crystal violet. The magnitude of MSC migration was evaluated by counting the number of migrated cells under a Nikon Eclipse 90i microscope at a magnification of 200×.

### Apoptosis staining

Annexin V-FITC/propidium iodide (PI) double staining was used to evaluate apoptosis by flow cytometry according to the manufacturer’s instructions as in a previous study [13]. Cells (1.0 × 10^6^) were collected and suspended in 500 μL binding buffer, and 5 μL annexin V-FITC and 5 μL PI were added to each sample and incubated in the dark for 15 min. The cell surface levels of phosphatidylserine in apoptotic cells were estimated quantitatively using flow cytometry (FACS LSRFortessa).

### Statistical analysis

Comparisons of parameters between two groups were made with an unpaired Student’s *t* test. Statistical analysis was carried out using SPSS 13.0 software (SPSS Inc., Chicago, IL). All data are expressed as the mean ± standard deviation (SD). *P* < 0.05 was regarded as a significant statistical difference.
